# Effect of Nitrogen Gas Post-Curing and Printer Type on the Mechanical Properties of 3D-Printed Hard Occlusal Splint Material

**DOI:** 10.3390/polym14193971

**Published:** 2022-09-22

**Authors:** Junichiro Wada, Kanae Wada, Mona Gibreel, Noriyuki Wakabayashi, Tsutomu Iwamoto, Pekka K. Vallittu, Lippo Lassila

**Affiliations:** 1 Department of Biomaterials Science, Turku Clinical Biomaterials Centre—TCBC, Institute of Dentistry, University of Turku, Itäinen Pitkäkatu 4B, 20520 Turku, Finland; 2 Department of Advanced Prosthodontics, Tokyo Medical and Dental University—TMDU, 1-5-45, Yushima, Bunkyo-ku, Tokyo 113-8510, Japan; 3 Department of Pediatric Dentistry/Special Needs Dentistry, Tokyo Medical and Dental University—TMDU, 1-5-45, Yushima, Bunkyo-ku, Tokyo 113-8510, Japan; 4 City of Turku Welfare Division, Oral Health Care, Puolalankatu 5, 20101 Turku, Finland

**Keywords:** 3D printing, mechanical property, degree of double bond conversion, microlayer structure, occlusal splint, post-curing, nitrogen gas

## Abstract

Although three-dimensional (3D) printing is clinically convenient to fabricate occlusal splints, it is still unclear how the post-curing method and the printer type can affect 3D-printed splints. This study aimed to evaluate the effect of stroboscopic post-curing at a nitrogen gas (N_2_) atmosphere versus post-curing in an air atmosphere, as well as the printer type (liquid crystal display (LCD) and digital light processing (DLP)) on the mechanical properties of a 3D-printed hard-type occlusal splint material. Flexural strength, flexural modulus, Vickers hardness number (VHN), fracture toughness, degree of double bond conversion (DC), 3D microlayer structure, water sorption, and water solubility were evaluated. The post-curing method significantly affected all evaluated properties except fracture toughness and 3D microlayer structure, while the printer type significantly affected all evaluated properties except flexural strength and flexural modulus. VHN and DC were significantly higher, and the smoother surface was noticeably obtained when printed by LCD printer and post-cured at an N_2_ atmosphere. The current results suggested that the post-curing method and the printer type would play a role in the mechanical properties of the evaluated material and that the combination of post-curing at an N_2_ atmosphere and LCD printer could enhance its mechanical properties and surface smoothness.

## 1. Introduction

Hard-type occlusal splints are oral appliances used for temporomandibular disorders treatment [1,2], prevention of tooth wear under severe sleep bruxism [3], and protection of mobile teeth due to severe periodontitis [4]. The use of hard-type occlusal splints for the above-mentioned treatments has a low risk of side effects and is clinically accepted [2,3]. Hard-type occlusal splint placement reduces muscular activity and distributes occlusal forces generated during teeth grinding and/or clenching, leading to the prevention of additional tooth wear and/or secondary occlusal trauma [5]. Conventionally, hard-type occlusal splints have been fabricated using heat-cured or auto-polymerizing polymethyl methacrylate (PMMA) [6]. The conventional occlusal splint fabrication needs a working cast made from an impression taken in the patient’s oral cavity and intraoral adjustments to gain ideal fitting and function. The above-mentioned fabrication process is time-consuming for dentists and patients, as well. In addition, the fitting accuracy of occlusal splints and their clinical performance are critically affected by several factors including residual monomer content, bubble formation, and dimensional change which may occur during the production process [7].

Three-dimensional (3D) printing has recently been used as an alternative to the conventional method for the fabrication of dental appliances including occlusal splints. 3D printing is one of the additive manufacturing technologies that uses two-dimensional (2D) data derived from the segmentation of 3D models produced using computer-aided design (CAD) and computer-aided manufacturing (CAM) systems to physically replicate 3D objects in a layer-by-layer method [8,9,10,11]. When compared to the conventional method, 3D printing is more time-efficient and cost-effective, and it also results in higher fitting accuracy [10,12] because of fewer errors with technical complications during multiple analog processing. Furthermore, 3D printing can constantly provide accurate thickness and shape of occlusal splints [8]. Therefore, the 3D printing method can lead to more predictable results than the conventional one [13].

Currently, various systems of 3D printing are clinically available. According to the printing process, the printing methods can be categorized into the following four types: (1) stereolithography printing, where a direct light illumination is projected through the bottom of a resin tray and prints the material (photopolymers) [14]; (2) inkjet printing, where the allocation of droplets of ink on a three-axis stage is followed by the printing [15]; (3) extrusion printing, where the warmed material is pushed through a nozzle and printed on a computer-controlled three-axis stage [16]; and (4) selective laser melting (SLM), where the material (powders) sintered region-specifically by the high temperature of the laser is coupled layer-by-layer generating a 3D structure with a computer-controlled movement of powder bed [17]. Among these methods, stereolithography printing is commonly used for occlusal splint fabrication [8,18]. In the stereolithography printing method, liquid crystal display (LCD) and digital light processing (DLP) are common as fast 3D printing processes for the fabrication of dental applications [19,20]. LCD printers generate a mask and block out the light from the light-emitting diode (LED) back panel using a liquid crystal display, while DLP printers project the image using a screen by reflecting the light [19]. Partially because DLP printers typically have higher light intensity than LCD printers, dental materials for 3D printing have been mainly developed for DLP 3D printers [21,22]. Meanwhile, previous studies indicated that the 3D printer type could affect the mechanical properties of 3D-printed objects [23,24]. However, the effect of printer type on 3D-printed occlusal splint materials is still unclear.

In order to finalize the polymerization process, the 3D-printed objects should be rinsed with a solvent to remove unpolymerized resin and then placed in a light polymerization chamber [25,26]. Several studies [27,28,29,30,31] that investigated the post-curing method for 3D printing using temporary prostheses and denture base materials suggested that the post-curing method would have an impact on the mechanical properties of those materials. A previous report indicated that stroboscopic post-curing at a nitrogen gas (N_2_) atmosphere could improve the degree of conversion of a 3D printing material for temporary restorations [27]. Although few studies have focused on the mechanical properties of materials used for 3D printing of occlusal splints [12,18], the effect of post-curing on their mechanical properties is poorly documented [32,33].

Therefore, the aim of this study was to evaluate the effect of the post-curing method and 3D printer type on the mechanical properties of a 3D-printed hard-type occlusal splint material. The following two null hypotheses were tested: (1) there would be no differences in the mechanical properties between 3D-printed hard-type occlusal splint materials post-cured with different post-curing methods; and (2) there would be no differences in the mechanical properties between 3D-printed hard-type occlusal splint materials printed by different printers.

## 2. Materials and Methods

### 2.1. Flexural Strength and Modulus Testing

**Figure 1:**
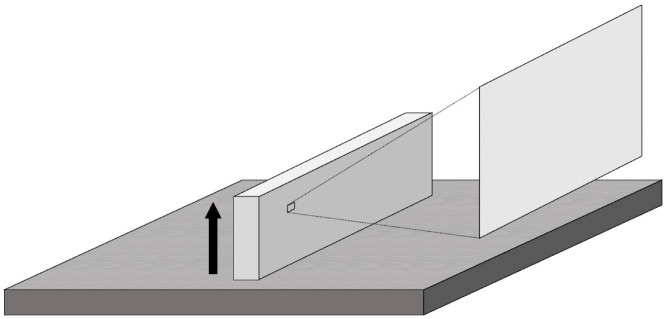
The printing direction (black arrow) and evaluated region for Vickers hardness number (VHN) and three-dimensional (3D) microlayer structure.

For flexural strength and modulus testing, 80 bar-shaped specimens (3.0 × 10.0 × 60.0 mm^3^) were 3D-printed with a layer thickness of 100 µm in a horizontal direction (Figure 1) using a photopolymerizing resin used for 3D-printed hard-type occlusal splint (KeySplint^®^ Hard, Keystone Industries GmbH, Singen, Germany). Half of the specimens (*n* = 40) were printed by a liquid crystal display (LCD) printer with 405 nm (Creo™ C5, PLANMECA OY, Helsinki, Finland), named as the Creo group, while the other half (*n* = 40) were printed by a digital light processing (DLP) printer with 385 nm (Asiga MAX™, SCHEU-DENTAL GmbH, Iserlohn, Germany), named as the Asiga group. The specimens were rinsed with isopropanol for 10 min in an ultrasonic cleaning unit (Quantrex^®^ 90, L&R Ultrasonics, Kearny, NJ, USA). Afterward, each group was divided into two equal subgroups based on the atmosphere during a stroboscopic post-curing with 2000 flashes on each surface (Otoflash G171, BEGO GmbH&Co, Bremen, Germany), as follows: post-curing at an N_2_ atmosphere or in the air atmosphere (*n* = 20/subgroup). For each subgroup, half of the specimens were aged in boiling deionized distilled water for 16 h before testing, while the other half were stored in the air atmosphere without any aging.

The flexural strength (MPa) and modulus (GPa) were evaluated at room temperature by a 3-point bending test across a 50 mm span at a crosshead speed of 5.0 mm/min using a universal testing machine (Model LRX; Lloyds Instruments Ltd., Hampshire, UK) with a load cell of which the capacity was 2500 N. Each test was detected to be finished when the reduction in the load reached 10% of the maximum load or when the deflection of the specimen reached 12 mm. Additionally, the number of specimens which were broken during the three-point bending test was recorded and the ratio of the broken specimens (%) was calculated for each group.

### 2.2. Surface Microhardness (Vickers Hardness)

For each subgroup, surface hardness measurement was performed on two randomly selected specimens’ fragments from both aged and non-aged subgroups after the three-point bending test. The Vickers hardness number (VHN) was measured on each selected specimen at 10 different regions using a Vickers hardness testing device (Duramin-5, Struers, Ballerup, Denmark) applying a 9.81 N force for 5 s. An indentation was created on each selected region of the specimen with a square-based pyramid diamond indenter. The diagonals of the pyramid impressed on the specimen were measured on a microscopic scale by the eyepiece operator of the testing device, and then VHN was calculated using the following equation:

VHN = 0.1891 × F/d^2^ (1)

where F is the indenting force (N) and d is the mean length of two diagonals of the squared base of indentation (mm).

### 2.3. Surface Polymerization (Degree of Double Bond Conversion; DC)

For five specimens which were randomly selected from each non-aged subgroup, the degree of double bond conversion (DC) was measured using a Fourier Transform Infrared (FTIR) spectrometer (Frontier FT-IR spectrometer, PerkinElmer, Llantrisant, UK). The unpolymerized material was measured as the control. The ratio of the absorbance intensity of the aliphatic C=C absorbance peak at 1640 cm^−1^ to the aromatic reference peak at 1610 cm^−1^ was detected in both unpolymerized material and selected specimens. The DC values were calculated as a percentage (%) using the following equation:

DC = 100 ∗ [1 − (C_alipha_/C_aroma_)/(U_alipha_/U_aroma_)] (2)

where C_alipha_ is the polymerized aliphatic peaks ratio; C_aroma_ is the polymerized aromatic peaks ratio; U_alipha_ is the unpolymerized aliphatic peaks ratio; and U_aroma_ is the unpolymerized aromatic peaks ratio.

### 2.4. Water Sorption and Solubility

Eight specimens were selected from each non-aged subgroup and used for testing the water sorption and solubility. The test started by drying the specimens in a vacuum desiccator containing freshly dried silica at 37 ± 1 °C for 22 h and then at 23 ± 1 °C for 2 h (the first drying procedure). The initial mass (M_1_) of each specimen was measured using a digital analytical balance (XS105; Mettler Toledo, Greifensee, Switzerland) to an accuracy of 0.1 mg. Then, the first drying procedure was continued until the mass decrease during a single day was less than 0.1 mg for all the specimens. After achieving a steady mass of each specimen and finishing the first drying procedure, the specimens were immersed in 40.0 mL of distilled water at 37 °C for 30 days. The mass of water-immersed specimens was measured 60 s after being removed from the water and carefully dried with absorbent paper at 1, 2, 3, 7, 14, 21, 28, and 30 days (M_2_) after immersion into the water. Finally, according to the same manner as the first drying procedure, the second drying procedure was continued until a consistent mass (M_3_) of each specimen was achieved. The water sorption and solubility were calculated in percentages (%) using the following equations:

Water sorption = 100 × (M_2_ − M_3_)/M_1_ (3)

Water solubility = 100 × (M_1_ − M_3_)/M_1_, (4)

where M_1_ is the initial mass of the specimen after storing it in a desiccator for 24 h (mg); M_2_ is the mass of the specimen after water immersion for 30 days (mg); and M_3_ is the constant mass of the specimen after the second drying cycle (mg).

### 2.5. Three-Dimensional Microlayer Structure

One specimen was randomly selected from each non-aged subgroup and further gold-sputtered in a sputtering device for the measurement of the 3D microlayer structure (width, length, and height of one structural unit) in micrometers (µm). The 3D microlayer structure was measured in 10 different regions (150 µm × 150 µm) on the surface of each selected specimen using a 3D optical profilometer (ContourGT-I, Bruker Nano, Inc., Tucson, AZ, USA). Additionally, the surface condition of the selected specimen was visually observed using a scanning electron microscope (SEM) (JSM-5500, JEOL Ltd., Tokyo, Japan).

### 2.6. Fracture Toughness (K_IC_)

Sixty-four 3D-printed bar-shaped specimens (8.0 × 4.0 × 40.0 mm^3^) were additionally fabricated and divided into groups in the same manner as described in Section 2.1. for fracture toughness (K_IC_) testing. Fracture toughness was measured using a single-edge notched bend (SENB) test. Each specimen was placed on a holder and then centrally notched using a double-sided diamond disk with 0.15 mm thickness (Komet, Brassler, Legmo, Germany). The length of the notch of each specimen was standardized to be 3.0 mm using a light microscope (Leica; Leica Microsystem GmbH, Wetzlar, Germany). Then, the polishing and sharpening of the notch were completed using a straight-edged razor blade, followed by a 3-point bending test across a 32 mm span at a crosshead speed of 1.0 mm/min with a universal testing machine. After testing, the notch length was measured three times using a light microscope, and the average of three measurements was calculated as the crack length on the fracture surface of each specimen (mm). The K_IC_ (MPa m^1/2^) was calculated using the following equation:

K_IC_ = [(P L)/(B W^3/2^)]f(x) √ 10^−3^ f(x) = 3x^1/2^[1.99 − x(1 − x)(2.15 − 3.93x + 2.7x^2^)]/[2(1 + 2x)(1 − x)^3/2^] and 0 < x < 1 with x = a/W(5)

where P is the maximum load (N); L is the span distance in millimeters (32.0 mm); B is the specimen width (mm); W is the specimen thickness or height (mm); x is a geometric function dependent on a/W; and a is the crack length (mm).

### 2.7. Statistical Analysis

The data from all evaluated properties except for the ratio of the broken specimens were statistically analyzed. The equality of variance was validated by the Levene test. A one-way ANOVA with a Tukey multiple comparison post hoc test was performed using statistical software (SPSS Statistics v28.0, IBM, Redmond, WA, USA) with the significance level set at 0.05. The ratio of the broken specimens was statistically compared using a Chi-squared test and Bonferroni correction. In addition, a three-way ANOVA was performed to detect the statistical significance of the effect of three factors (post-curing method, printer type, and aging in boiling water), independently, on the mechanical properties evaluated both in aged and non-aged specimens (flexural strength, flexural modulus, VHD, and fracture toughness), while a two-way ANOVA was performed to detect those of two factors (post-curing method and printer type), independently, on the mechanical properties evaluated only in non-aged specimens (DC, 3D microlayer structure, water sorption, and water solubility).

## 3. Results

The *p* values of multiple-way ANOVA are shown in Table 1. The two-way ANOVA showed that the post-curing method and the printer type significantly affected the DC, 3D microlayer structure, water sorption, and solubility (*p* < 0.001), while the three-way ANOVA showed that the post-curing method, the printer type, and the aging in boiling water significantly affected the flexural strength, flexural modulus, VHN, and fracture toughness (*p* < 0.001). On the other hand, no significant effect was found in the post-curing method on the fracture toughness (*p* = 0.069), 3D microlayer structure (*p* = 0.335, 0.127, and 0.907, for width, length, and height, respectively), and water solubility (*p* = 0.968). In addition, the effect of the printer type on the flexural strength (*p* = 0.323) and flexural modulus (*p* = 0.220) was not significant.

**Figure 2:**
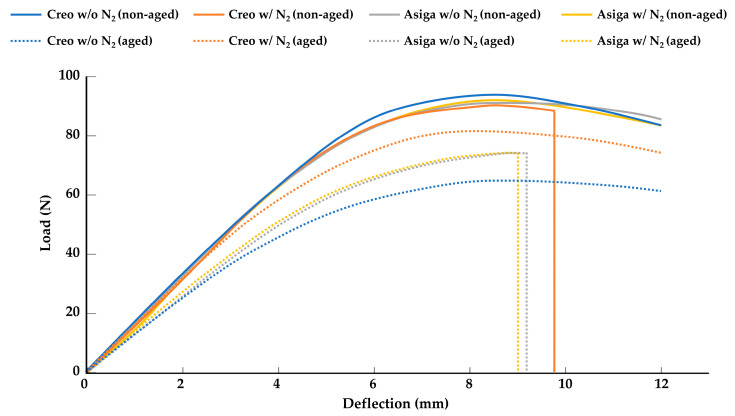
Typical load deflection curve for each group. Note that all specimens are broken in aged Asiga subgroups while no specimen are broken in non-aged Asiga subgroups, and a positive effect of the post-curing at a nitrogen gas (N_2_) atmosphere is found only in aged Creo subgroups.

The mean values and standard deviations of the flexural strength, flexural modulus, VHN, and fracture toughness are shown in Table 2. Generally, the aging in boiling water resulted in lower flexural strength, flexural modulus, and fracture toughness compared to the specimens stored in the air atmosphere without any aging. Only in the Creo groups, post-curing at an N_2_ atmosphere significantly enhanced the flexural strength and flexural modulus of the groups aged in boiling water (*p* < 0.001). However, the effect of the post-curing at an N_2_ atmosphere on the flexural strength, flexural modulus, and the ratio of broken specimens was not significant in the non-aged specimens. Figure 2 represents the typical load-deflection curve for each aged and non-aged group. Regardless of the post-curing method, the ratio of broken specimens in Asiga groups with aging was significantly higher than that in Asiga groups without aging, while there was no significant difference in the ratio of broken specimens between Creo groups with and without aging. The Asiga groups showed significantly higher fracture toughness than the Creo groups (*p* < 0.001). The post-curing at an N_2_ atmosphere negatively affected the fracture toughness only in the non-aged specimens for the Asiga groups. The Creo groups showed significantly higher VHN than the Asiga groups (*p* < 0.001). Regardless of the printer type, the post-curing at an N_2_ atmosphere significantly enhanced the VHN (*p* < 0.001). On the other hand, with the exception of the Asiga subgroups post-cured at an N_2_ atmosphere, the aging in boiling water had no significant effect on the VHN.

**Figure 3:**
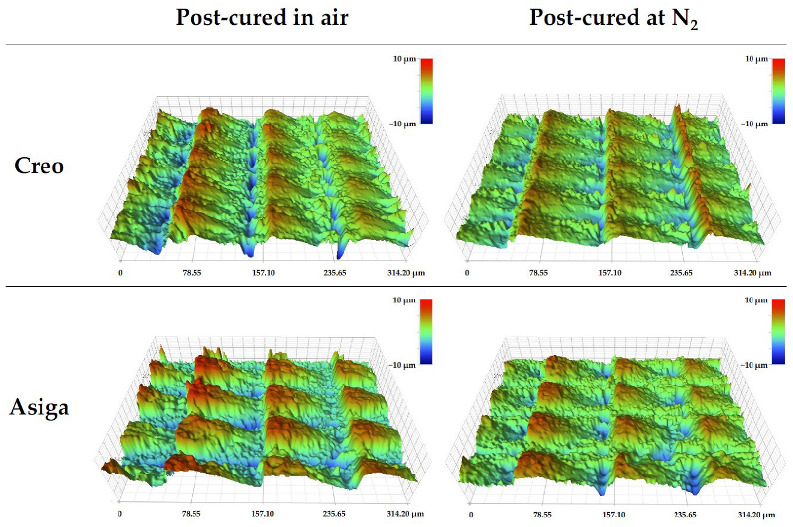
3D surface images of typical specimens obtained by the optical profilometer. Note the shallower grooves between microstructures in the Creo specimen post-cured at an N_2_ atmosphere compared to other specimens.

**Figure 4:**
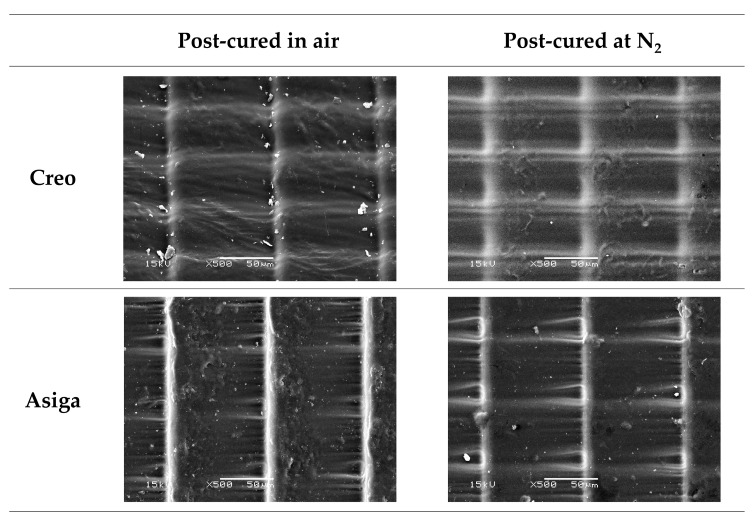
Scanning electron microscope (SEM) images (×500 at 15kV) of microlayer structures on the surface of typical specimens. Note the smoother surface on the Creo specimen post-cured at an N_2_ atmosphere compared to other specimens.

The mean values and standard deviations of DC, 3D microlayer structure, water sorption, and solubility are shown in Table 3. Regardless of the post-curing method, the Creo groups showed higher DC than the Asiga groups, and a significant difference was found between the Creo and Asiga subgroups post-cured at an N_2_ atmosphere (*p* < 0.001). In both Creo and Asiga groups, the post-curing at an N_2_ significantly enhanced the DC (*p* < 0.001). The typical images of the profilometer and SEM are shown in Figure 3 and Figure 4. The post-curing method showed no significant effect on the 3D microlayer structure, while the size of a 3D microlayer structural unit (width, length, and height) in the Creo groups was significantly smaller than that in the Asiga groups (*p* < 0.001). The SEM images showed that a smoother surface was obtained in the Creo subgroup post-cured at an N_2_ atmosphere when compared to the other subgroups as well as the optical profilometer image.

**Figure 5:**
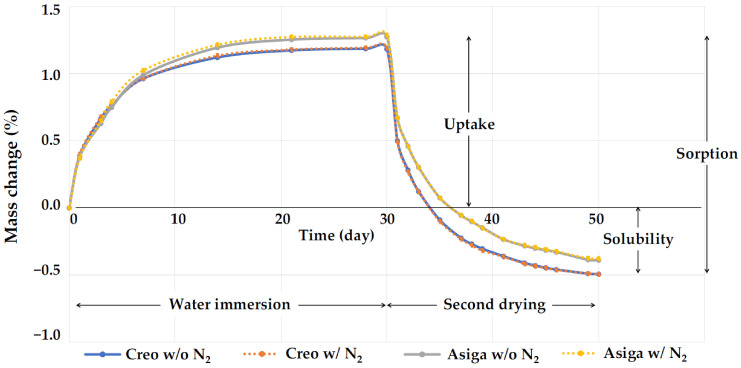
The representative plots of mass changes (%) against time during the water immersion and the second drying procedure. Note the saturation 14 days after water immersion and completion of drying 20 days after drying began in all groups.

The representative plots of mass changes (%) against time during the water immersion and the second drying procedure are shown in Figure 5. In all groups, saturation was achieved 14 days after water immersion. With the second drying procedure, although the Creo subgroups started to lose water faster than the Asiga subgroups at the beginning, completion of drying was achieved 20 days after drying began in all subgroups. The water sorption for subgroups post-cured at an N_2_ atmosphere was significantly higher than that for those post-cured in the air atmosphere (*p* = 0.024 and 0.012 for Creo and Asiga subgroups, respectively), while no significant difference was found in the water solubility in both Creo and Asiga subgroups (*p* = 0.998 and 0.995 for Creo and Asiga subgroups, respectively).

## 4. Discussion

This study demonstrated the effect of the post-curing method and printer type on the mechanical properties, including the flexural strength, flexural modulus, surface microhardness (VHN), fracture toughness, water sorption, and water solubility in addition to the surface polymerization (DC) and 3D microlayer structures in a 3D-printed hard-type occlusal splint material. The overall results rejected the two null hypotheses and revealed that stroboscopic post-curing at an N_2_ atmosphere enhanced the mechanical properties and DC of the 3D-printed hard-type occlusal splint material and that the printer type played a significant role in their mechanical properties, DC, and 3D microlayer structure.

The stroboscopic post-curing at an N_2_ atmosphere, which was applied in this study, has been reported to be more effective to obtain a higher DC on the surface of 3D-printed temporary crowns and bridges material than post-curing using ultraviolet light or LED in the air atmosphere [27]. Consistently, in this study, post-curing at an N_2_ atmosphere significantly enhanced DC and VHN. In addition—though limitedly in Creo subgroups—the surface condition was noticeably smoother in specimens post-cured at an N_2_ atmosphere than in those post-cured in the air atmosphere (Figure 3 and Figure 4). An N_2_ atmosphere might prevent the formation of an oxygen inhibition layer so that the post-polymerization on the surface of specimens could be consequently enhanced. On the other hand, the effect of the post-curing method on the fracture toughness of the specimens was not significant.

In this study, the SENB test was used to assess fracture toughness. The values of fracture toughness obtained using this test are mainly affected by the inner stress of the specimens, possibly leading to the explanation that the post-curing method employed to finalize the polymerization of only the surface layer of specimens could not significantly affect the fracture toughness in this study. Additionally, it should be mentioned that the flexural modulus and VHN of specimens post-cured at an N_2_ atmosphere were lower when compared to conventional heat-cured and auto-polymerizing PMMAs, while their fracture toughness was equal to them or higher [34]. Therefore, further studies are required to clarify the clinical acceptance of the effect of post-curing at an N_2_ atmosphere on the mechanical properties of the 3D-printed occlusal splint materials.

In this study, two different printers, an LCD printer (Creo) and a DLP printer (Asiga), were used to fabricate the specimen, although the manufacturer’s guideline of the evaluated material recommends using DLP printers. A previous study [20] investigated the effect of printer type on the mechanical properties of materials for 3D-printed temporary crowns and bridges and came to the conclusion that the materials for which DLP printers were recommended to be used could achieve acceptable mechanical properties even when printed by LCD printers. In this study, although the layer thickness was set at a fixed value (100 µm), a microlayer structural unit in the Creo subgroups had a lower height, suggesting that a smoother surface was obtained, and its size was more stable than in the Asiga subgroups. The difference in image projection methods between LCD and DLP printers might explain this variation. Basically, in DLP printers, the microlayer structures might be affected by the location of the specimens on the screen due to the diffusion of light during its reflection. In contrast, it has been reported that DLP printers had higher dimensional accuracy than LCD printers when the printed objects were bigger [35], indicating that the dimensional stability of the microlayer structures should not be linked to the overall dimensional accuracy of the printed objects. Regardless of the post-curing method, VHN and DC in the Creo subgroups were higher than those in the Asiga subgroups. A previous study evaluating the mechanical properties of a 3D-printed orthodontic aligner material reported that LCD printers provided a higher surface hardness of the printed objects than DLP printers [24]. In this study, a well-polymerized surface with a smooth and stable microlayer structure of the specimens fabricated by the Creo printer with LCD could be the reason for the higher VHN.

Meanwhile, regardless of the post-curing method, the Asiga subgroups displayed lower water sorption and solubility than the Creo subgroups (Table 2). Additionally, in both Creo and Asiga subgroups, the water sorption of specimens post-cured at an N_2_ atmosphere was less than those post-cured in the air atmosphere. Furthermore, the water sorption and solubility of the splint material evaluated in this study were equal to or higher than those of conventional and CAD-CAM milled PMMA materials used for hard-type occlusal splints [34]. Thus, the current study revealed that the water sorption and solubility of the evaluated material would be clinically acceptable regardless of the post-curing method and printer type.

Aging in boiling water is one of the common aging methods for testing dental materials [36]. The purpose of this aging method is to accelerate aging and evaluate the effect of hydrolytic and thermal breakdown. Thus, it has been reported to be efficient to assess material stability against distress [37,38]. In this study, aging in boiling water led to critically lower flexural strength, flexural modulus, and fracture toughness of the tested specimens, while it had no significant impact on their VHN. It is interesting to note that post-curing at an N_2_ atmosphere significantly enhanced the flexural strength and modulus of specimens aged in boiling water only in the Creo subgroups. This finding suggested that post-curing at an N_2_ atmosphere could enhance the mechanical properties of the evaluated material when printed by an LCD printer. However, because the aging condition used in this study was different from the real clinical situation, there is a need for further studies to clinically assess the mechanical properties of 3D-printed hard-type occlusal splints over time in real patients.

The fixed layer thickness, the shape of the specimens, which differs from the clinically used arch-shaped ones, and the static mechanical stress applied during testing are considered as limitations of this study. Additionally, intraoral scanners have been becoming more common for clinical applications [39]. To maximize the strong point of digital technologies for dentistry, “full digital procedure” without any intermediate analog materials, such as impression materials and/or dental stones for working casts, should be necessary. As previously mentioned, further studies are necessary to clarify the clinical performance of 3D-printed hard-type occlusal splints, including the fabrication of splints directly from digital impressions of dental arches using intraoral scanners. Finally, since the chemical composition of the evaluated material was not provided by the manufacturer, it was difficult to come to a conclusion on whether there were some differences in chemical composition between the evaluated material and the other materials intended for the same purpose; therefore, the generalization of our findings for all of the 3D-printed hard-type occlusal splints might not be applicable.

## 5. Conclusions

Within the limitations of this study, the following can be concluded: (1) The post-curing method and the printer type played a role in the mechanical properties of the evaluated 3D printed hard-type occlusal splint material; and (2) The combination of printing by a liquid crystal display printer and the post-curing at a nitrogen gas atmosphere can enhance the mechanical properties and surface smoothness of 3D printed hard-type occlusal splints and micrify their distress with aging in boiling water.

## Figures and Tables

**Table 1:** *p* values of multiple-way ANOVA statistical analysis for the evaluated mechanical properties.

Variable	Flexural Strength	Flexural Modulus	Vickers Hardness Number (VHN)	Fracture Toughness	Degree of Double Bond Conversion (DC)	Three-Dimensional (3D) Microlayer Structure			Water Sorption	Water Solubility
						Width	Length	Height		
Post-curing method	<0.001	<0.001	<0.001	0.069	<0.001	0.335	0.127	0.907	<0.001	0.968
Printer type	0.323	0.220	<0.001	<0.001	<0.001	<0.001	<0.001	<0.001	<0.001	<0.001
Aging in boiling water	<0.001	<0.001	<0.001	<0.001	-	-	-	-	-	-

*p* < 0.05 is significant.

**Table 2:** Mean value and standard deviation of flexural strength, flexural modulus, ratio of broken specimens during three-point bending test, VHN, and fracture toughness, and results of one-way ANOVA statistical analysis and Chi-squared test.

Printer Type	Post-Curing	Aging in BW	Flexural Strength (MPa)		Flexural Modulus (GPa)		Broken Specimens (%)		VHN		Fracture Toughness (MPa m^1/2^)	
				^#^		^#^		^##^		^#^		^#^
Creo	SS	-	92.5 ± 3.1	^ac^	2.21 ± 0.11	^acd^	30	^a^	12.5 ± 0.4	^abf^	2.22 ± 0.13	^ac^
		+	66.3 ± 3.2	^b^	1.70 ± 0.14	^be^	40	^ab^	12.1 ± 0.4	^abd^	0.70 ± 0.06	^bg^
	SS + N_2_	-	90.6 ± 4.0	^c^	2.13 ± 0.13	^ac^	60	^ab^	15.7 ± 0.5	^c^	2.28 ± 0.12	^acf^
		+	80.5 ± 1.5	^d^	2.11 ± 0.07	^ac^	40	^ab^	15.3 ± 0.5	^c^	0.69 ± 0.05	^bg^
Asiga	SS	-	92.1 ± 2.8	^ac^	2.30 ± 0.09	^ad^	0	^a^	11.8 ± 0.6	^bd^	2.59 ± 0.11	^d^
		+	73.6 ± 1.8	^e^	1.79 ± 0.05	^bef^	100	^b^	11.6 ± 0.4	^d^	0.85 ± 0.05	^eg^
	SS + N_2_	-	92.8 ± 1.8	^ac^	2.32 ± 0.10	^ad^	0	^a^	13.3 ± 0.5	^e^	2.39 ± 0.13	^cf^
		+	73.8 ± 1.5	^e^	1.86 ± 0.06	^ef^	100	^b^	12.8 ± 0.5	^af^	0.83 ± 0.05	^beg^

SS: stroboscope; N_2_: nitrogen gas; VHN: Vickers hardness number; and BW: boiling water. -: Without aging (non-aged group); and +: with aging (aged group). ^#^ Same superscripted letters indicate groups not statistically significantly different when compared by one-way ANOVA and post hoc analysis with Tukey multiple comparisons (*p* > 0.05). ^##^ Same superscripted letters indicate groups not statistically significantly different when compared by Chi-squared test and Bonferroni correction (*p* > 0.05).

**Table 3:** Mean value and standard deviation of DC, 3D microlayer structure, water sorption, and water solubility, and the results of one-way ANOVA statistical analysis.

Printer Type	Post-Curing Method	DC (%)		3D Microlayer Structure (μm)						Water Sorption (%)		Water Solubility (%)	
				Width		Length		Height					
Creo	SS	64.7 ± 6.4	^a^	51.2 ± 0.4	^a^	97.7 ± 0.3	^a^	7.5 ± 1.2	^a^	1.685 ± 0.004	^a^	0.495 ± 0.020	^a^
	SS + N_2_	92.3 ± 4.5	^b^	51.3 ± 0.3	^a^	97.6 ± 0.4	^a^	7.3 ± 1.5	^a^	1.696 ± 0.006	^b^	0.496 ± 0.036	^a^
Asiga	SS	56.7 ± 6.2	^a^	62.0 ± 0.5	^b^	100.6 ± 1.3	^b^	13.4 ± 0.8	^b^	1.664 ± 0.003	^c^	0.383 ± 0.006	^b^
	SS + N_2_	75.4 ± 4.5	^c^	62.3 ± 0.7	^b^	99.7 ± 1.5	^b^	13.7 ± 1.0	^b^	1.675 ± 0.009	^d^	0.379 ± 0.024	^b^

DC: Degree of double bond conversion; SS: stroboscope; and N_2_: nitrogen gas. Same superscripted letters indicate groups not statistically significantly different when compared by one-way ANOVA and post hoc analysis with Tukey multiple comparisons (*p* > 0.05).

## Data Availability

The data presented in this study are available on reasonable request from the corresponding author.
